# Experimental Investigation on the Machinability Improvement in Magnetic-Field-Assisted Turning of Single-Crystal Copper

**DOI:** 10.3390/mi13122147

**Published:** 2022-12-04

**Authors:** Xian Wu, Yu Zhou, Congfu Fang, Laifa Zhu, Feng Jiang, Ke Sun, Yuan Li, Yiyang Lin

**Affiliations:** 1 College of Mechanical Engineering and Automation, Huaqiao University, Xiamen 361021, China; 2 Institute of Manufacturing Engineering, Huaqiao University, Xiamen 361021, China; 3 Xiamen Tongan Vocational Technology School, Xiamen 361100, China

**Keywords:** single-point diamond turning, magnetic field, magnetoplasticity effect, chip morphology, Lorentz force

## Abstract

The single-point diamond-turning operation is a commonly used method for ultra-precision machining of various non-ferrous materials. In this paper, a magnetic field was introduced into a single-point diamond-turning system, and magnetic-field-assisted turning experiments were carried out. The results revealed that the magnetic field affects the metal-cutting process in the form of the cutting force, chip morphology, and surface quality. Compared with traditional turning, magnetic-field assisted turning increases the cutting force by 1.6 times, because of the additional induced Lorentz force, and reduces the cutting-force ratio and friction coefficient on the rake surface by 16%, with the improved tribological property of the tool/chip contact-interface. The chip morphology in the magnetic-field-assisted turning shows the smaller chip-compression ratio and the continuous side-morphology. With the magnetoplasticity effect of the metal material and the friction reduction, magnetic-field-assisted turning is helpful for improving metal machinability and achieving better surface-quality.

## 1. Introduction

Single-point diamond turning (SPDT) has been extensively applied in many precision and ultra-precision machining fields, for example, optical lens, optical reflector, disk base, etc. It can achieve excellent dimension-accuracy and good surface-roughness on the nanoscale, by using natural-diamond cutting tools [1,2]. The high-precision parts made of nonferrous materials, such as various single-crystal and polycrystalline metals, are very suitable for production with SPDT [3,4]. SPDT has been a research hotspot in the mechanical-machining field for a long time. As a single-crystal metal, single-crystal copper is commonly used to produce optical reflectors and various substrate materials, owing to its good electrical and thermal conductivity and chemical catalytic-performance, as well [5]. Lee et al. [6] investigated the surface-quality anisotropy on machined surfaces in the diamond cutting of single-crystal copper, and the best machining-direction was suggested. The effect of machining parameters in the nanocutting of a single-crystal-copper workpiece was studied by Zhang et al. [7], who found the depth of cut showed a significant influence on the defect layer. Because of its softness feature, single-crystal copper is easy to cut, but relatively difficult to grind, due to the blockage of the grinding wheel. Hence, SPDT becomes one of the ideal and commonly used methods for ultra-precision machining.

Recently, the energy-field-assisted machining methods have attracted significant attention, in areas such as laser-assisted cutting, ultrasonic-vibration-assisted cutting and magnetic-field-assisted cutting [8,9,10]. The primary objective of energy-field-assisted cutting is to obtain the “1 + 1 > 2” effect, to further improve the machining quality. The magnetic-field-assisted cutting is a novel energy-field-assisted machining method. In 1976, Muju et al. [11,12] and Kumagai et al. [13] found that the external magnetic-field helped to decrease the cutting-tool wear. Therefore, this application of the magnetic field to the metal-cutting field has been developed into two branches. First, the magnetic field was used to magnetize the cutting tool to improve its material microstructure and cutting performance [14,15]. Then, Mansori et al. [16,17] introduced an additional magnetic-field action into the metal-cutting process, and found magnetic-field action can change metal-cutting mechanics and obtain tribological interface-modifications on tool–chip contact. The results revealed that magnetic-field action in the metal-cutting process is effective for reducing tool wear. Mkaddem et al. [18] found that with an increase of magnetic intensity in magnetic-field-assisted cutting, the shear angle increased, and the contact length and friction coefficient of tool–chip contact decreased. Alireza et al. [19] performed magnetic-field-assisted cutting experiments, and found that it achieved an approximately 94% decrease in flank wear and a 66% reduction in cutting force. Yip et al. systematically investigated the advantages of magnetic-field-assisted cutting, including tool-life enhancement, tool-tip-vibration reduction, material swelling and recovery reduction [20,21,22,23]. From this literature, it is concluded that the magnetic field is a promising energy-field-assisted machining method with various advantages, but the essential cutting mechanisms are inadequately understood at present.

To verify this novel energy-field-assisted machining method and investigate the influence of magnetic-field action during the metal-cutting process, magnetic-field-assisted turning experiments were conducted on single-crystal copper with (111) crystal plane. The actual magnetic-distribution in the cutting zone after introducing an external magnetic-field was analyzed. Then, the cutting force and cutting-force ratio in magnetic-field-assisted turning and the obtained chip and surface quality were compared with conventional turning-processes, to analyze the relevant cutting mechanisms.

## 2. Experimental Procedure

**Figure 1:**
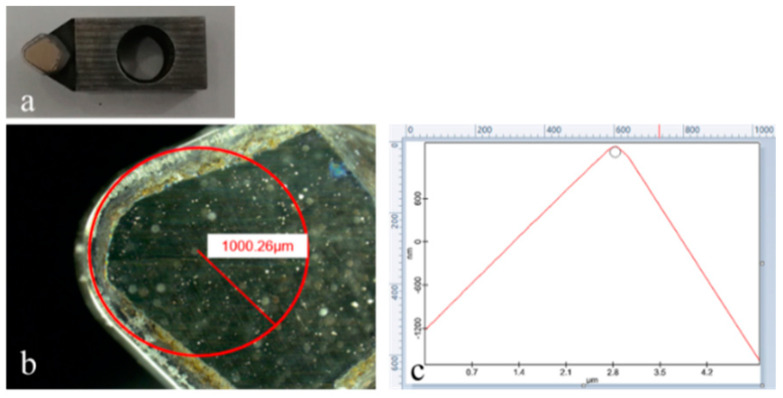
Single-crystal-diamond tool, (**a**) overall view, (**b**) tool nose, (**c**) cutting edge.

Single-crystal copper with (111) crystal plane was applied as a workpiece material in this work. In line with the dimensions of the machine-tool worktable, workpiece samples were pre-machined with the sizes of Φ150 × 25 mm. Single-crystal diamond is the perfect material for the cutting tool, due to its outstanding properties such as ultra-high hardness, low friction-coefficient and a cutting edge of high sharpness. The used single-crystal-diamond tool was prepared by Shanghai Suporhard Tools Co., Ltd. The single-crystal-diamond tool exhibits the rake- and flank-angle parameters of 0° and 5°, respectively, and its tool nose-radius was 1 mm, as shown in Figure 1. Roughness of tool surface greatly affects the friction properties between the tool and workpiece surface during the cutting process. Roughness of rake and flank surface were assessed as approximately 1.46 nm and 104.28 nm, respectively using the atomic force microscope (Alpha 300RA, WITec, UIm, Germany). As shown in Figure 1c, the edge radius of this used single-crystal-diamond tool was measured to approximately 115 nm.

**Figure 2:**
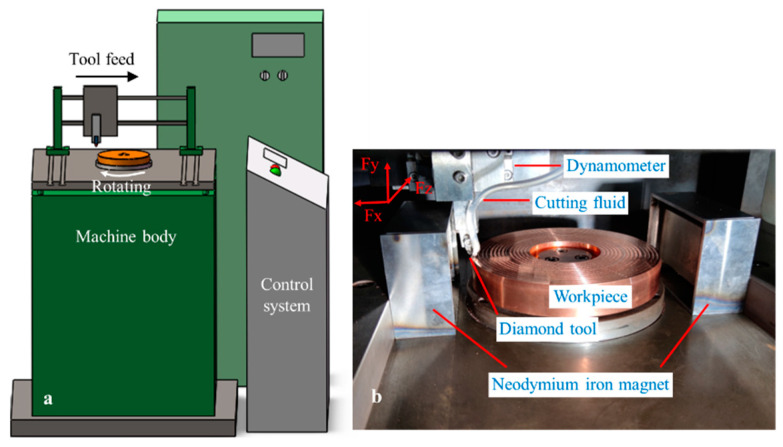
Single-point diamond-turning experiment, (**a**) machine tool, (**b**) magnetic-field-assisted turning.

Single-point diamond-turning experiments were performed with a self-developed vertical machine-tool which is specially designed for ultra-precision cutting, as shown in Figure 2. The main structures of this machine tool include the cast iron base, linear motor, hydrostatic bearing-spindle and CNC control system. The machine can provide a highest rotating-speed of 12,000 rpm, with a position precision of ±2 μm. The end-face runout of the spindle is less than 2 μm, with the vertical structure of the machine tool. As shown in Figure 2b, to realize magnetic-field-assisted single-point diamond face turning, two permanent magnets with different polarities were installed on the machine worktable. The magnets are made of neodymium iron with three dimensions of 100 × 50 × 20 mm. A magnetic-isolation cover fixture made of 45 steel was used to avoid the interference of the magnetic field on the linear motor. It can shield more than 96% magnetic intensity by testing with a tesla meter. Only one direction is opened to introduce the magnetic field into the workpiece and cutting zone. The remanence and coercive force were 1.18 T and 880 KA/m, respectively. The average magnetic-flux density is 0.38 T, and the distance between the two permanent magnets is 190 mm.

In line with the previous turning experiments, the cutting speed in this work was altered from 3.2 to 9.6 m/s, the depth of cut was varied from 4 to 12 μm, and five levels of feed rate were applied in single-factor experiments, as listed in Table 1. Before the experiments, a workpiece sample was pre-processed to guarantee surface flatness, with a PCD tool. Based on the previous test, industrial alcohol was used as the cutting fluid during the turning experiments. The cutting force was recorded online with the dynamometer (9119AA2, Kistler, Switzerland). After the experiments, the surface roughness and morphology were inspected, using a 3D optical-surface-profiler (New View 7300, Zygo, CT, USA). The chip morphology was observed using the electron scanning microscope (SEM, Phenom Pro, The Netherlands).

## 3. Results and Discussions

### 3.1. Magnetic-Field-Distribution Analysis

**Figure 3:**
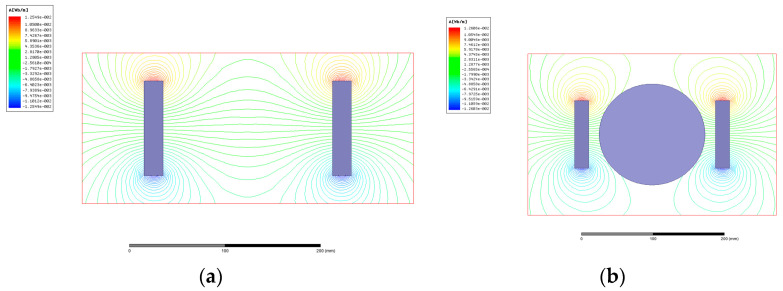
Magnetic-induction-line distribution comparison (left is N pole, right is S pole), (**a**) without workpiece, (**b**) with workpiece.

To research the influence of magnetic-field action on the metal-cutting process, the magnetic-field distribution is simulated with the software Maxwell. The corresponding simulation-model and boundary conditions are consistent with the magnetic-field-assisted turning. The magnet is neodymium iron N35H with same remanence and coercive force as the cutting experiments. The magnetic-induction-line distribution with and without the workpiece is shown in Figure 3. Before putting in the workpiece, the magnetic-induction line in the zone between the two permanent magnets is even, from the N pole to the S pole. In comparison, it is found that the magnetic-induction line exhibits no obvious variation with and without the workpiece. With the magnetic-induction-intensity measurement at five different positions on the workpiece surface, the results also indicate that the magnetic-induction intensity has no variety after the workpiece placing.

**Figure 4:**
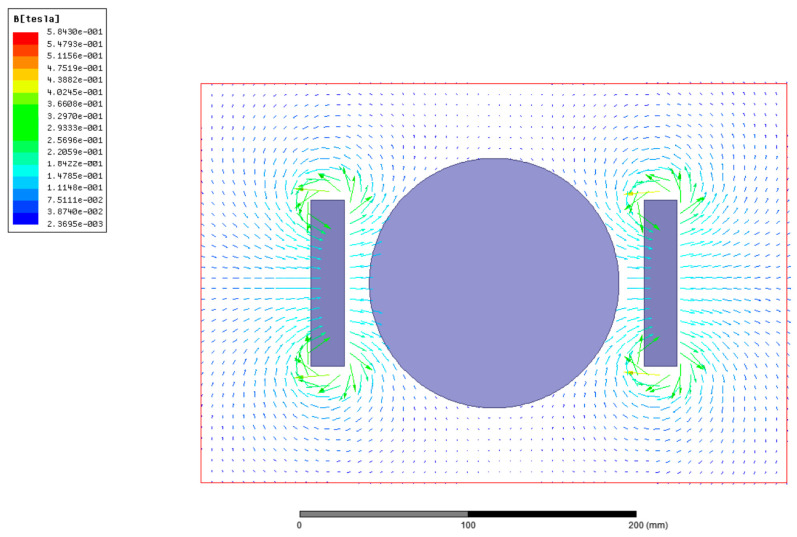
Magnetic-induction intensity distribution.

Magnetic-induction intensity distribution in the cutting zone is shown in Figure 4. The magnetic-induction line from N to S pole is vertical to the workpiece axial-direction. It is found that the even magnetic-field, which is formed by the neodymium iron magnet, can cover the whole scope of the single-crystal-copper workpiece. The magnetic field is introduced into the cutting zone, to analyze the effect on the cutting process.

### 3.2. Cutting Force and Friction Coefficient

**Figure 5:**
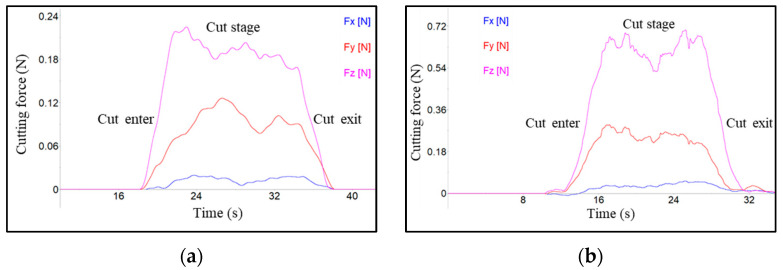
Cutting-force-signal comparison (*a_p_* = 10 μm, *f_r_* = 5.6 μm/r, *v* = 6.4 m/s), (**a**) cutting-force-signal without magnetic field, (**b**) cutting-force-signal with magnetic field.

Figure 5 shows the cutting-force-signal waveform that was recorded with and without the magnetic field in the turning process. The cutting-force directions in the turning experiments are illustrated in Figure 2b; among the three cutting-force components, tangential force, *Fz*, is the largest, second is the normal force, *Fy*, and the radial force, *Fx*, becomes the smallest. The depth of cut (4–12 μm) is much smaller than the tool-nose radius (1000 μm). Only the partial tool-nose assists in the metal-cutting process. This is the reason why the effective tool-cutting edge angle is very low, and is calculated to be less than 0.7°. Hence, the main components of the resultant force are distributed in a tangential direction, *Fz*, and a normal direction, *Fy*. The component of the resultant force in a radial direction, *Fx*, is very small, and close to zero, and it is difficult to acquire an accurate value. Hence, the subsequent analysis of the cutting force is mainly focused on the tangential and normal force. By comparison, it is found that the cutting-force waveform characteristics of magnetic-field-assisted and conventional turning, are similar. The cutting-force-signal waveform can be divided into three stages: the cut-enter stage, with rapidly increasing force, the cut stage, with stable force, and the cut-exit stage. with decreasing force. The average value of the cutting force in the stable stage was employed as the cutting-force result.

**Figure 6:**
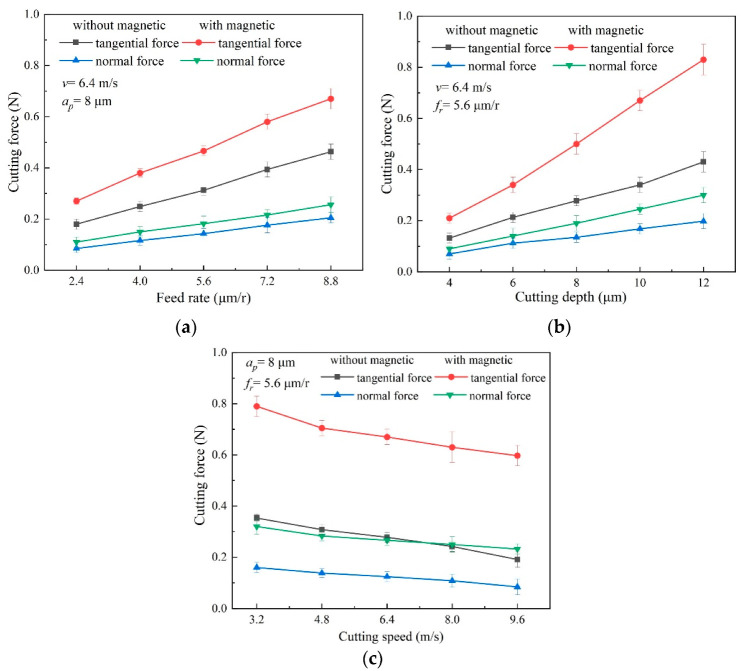
Effect of magnetic-field action on cutting force, (**a**) variation of cutting force with feed rate, (**b**) variation of cutting force with depth of cut, (**c**) variation of cutting force with cutting speed.

The cutting-force results under different machining parameters are shown in Figure 6. By comparison, the obtained cutting-force exhibits a similar variation trend, both in magnetic-field-assisted and traditional turning, under different machining parameters. Figure 6a exhibits the cutting-force curve varying with the tested-feed rate. While the feed rate rises from 2.4 to 8.8 μm/r, the tangential and normal cutting-force in conventional turning go up from 0.18 N and 0.09 N to 0.46 N and 0.21 N, respectively, and they increase from 0.27 N and 0.11 N to 0.67 N and 0.26 N respectively, in magnetic-field-assisted turning. It is found that, although both tangential and normal force increase along with the rising feed-rate, the discrepancy between tangential and normal cutting-force increases as well, at the same time. When *f_r_* = 2.4 μm/r, the discrepancies are 0.09 N and 0.16 N without and with the magnetic field, respectively, and increase to 0.25 N and 0.41 N, respectively, when *f_r_* = 8.8 μm/r. This indicates that the tangential cutting-force exhibits a higher increment than the normal force. Since the depth of cut rises from 4 to 12 μm, the cutting force exhibits an almost linear increase, as shown in Figure 6b. The same results are observed for the discrepancy between the tangential and normal cutting-force, which increases along with the rising depth of the cut. A possible reason is that the effective tool-cutting edge angle becomes larger, along with both the rising depth of cut and the feed rate. This reduces the component force in an axial direction, and then results in the larger discrepancy between tangential and normal cutting-force. As shown in Figure 6c, both tangential and normal cutting-force gradually decrease, along with the rising cutting-speed. 

**Figure 7:**
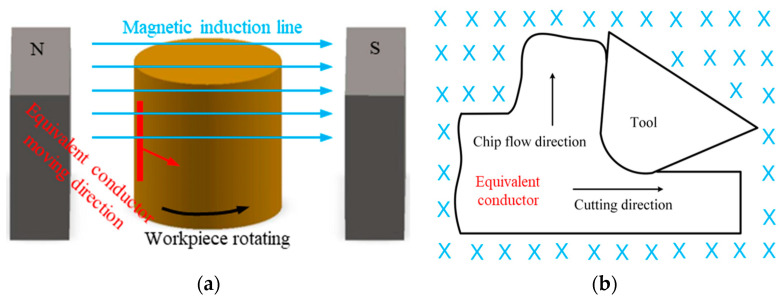
Equivalent conductor cuts the magnetic-induction line in the cutting process, (**a**) workpiece rotating in magnetic field, (**b**) equivalent-conductor-cut magnetic line.

**Figure 8:**
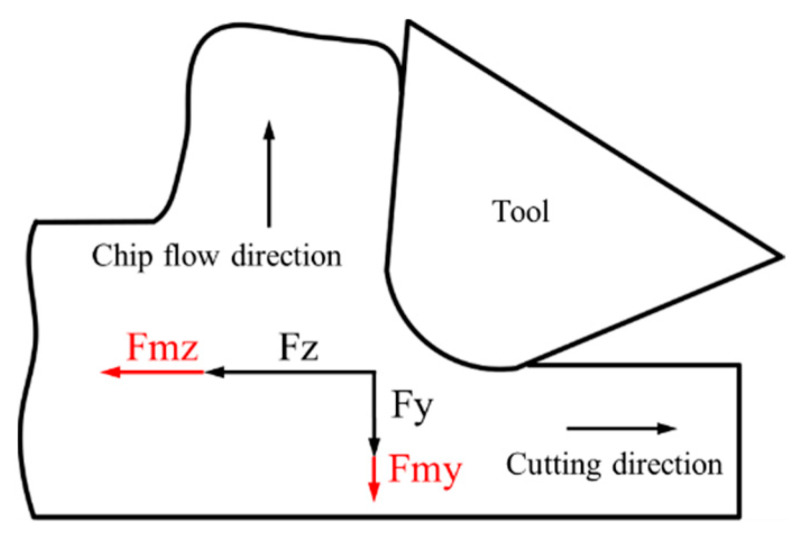
The induced Lorentz force.

From the cutting-force comparison between magnetic-field-assisted turning and conventional turning, it is clear that the cutting force in magnetic-field-assisted turning is relatively larger than that of traditional turning. According to the statistical data of the cutting-force results, tangential and normal cutting-force in magnetic-field-assisted turning are an average of 1.9 and 1.6 times, respectively, that of traditional turning. Single-crystal copper is a good conductor, while the copper workpiece rotates in the magnetic-field zone during the turning process; this can be equivalent to the case in which a conductor in a closed circuit cuts the magnetic-induction line in the introduced magnetic-field zone [24,25], as shown in Figure 7. According to the electromagnetic induction principle, the eddy current will be induced inside the copper workpiece, and the eddy current obeys the following equation:

*E* = *BLv* (1)

where *E* represents the eddy current, and *B* represents the magnetic-flux density, *L* represents the conductor length and *v* represents the cutting velocity. The induced eddy-current will produce an additional Lorentz force, *F_m_*, on the workpiece [18], which obeys the following equation:

*F_m_* = *BEL* (2)

where *F_m_* represents the Lorentz force and *E* represents the induced eddy-current. The direction of this Lorentz force is exactly opposite to the movement direction of the equivalent conductor to prevent its relative motion, and the value of this Lorentz force is proportional to the length of the equivalent conductor. As shown in Figure 8, the Lorentz force, *Fmz*, that is induced by the cutting movement, is added to the tangential force, and the Lorentz force, *Fmy*, which is induced by the chip-flow movement, is added to the normal force during the cutting process. Due to the relatively larger area of the workpiece material, compared with that of the produced chips during the cutting process, the induced Lorentz force, *Fmz*, in a tangential direction is relatively larger than the Lorentz force, *Fmy*, in the normal direction. This reason explains exactly the larger cutting-force in magnetic-field-assisted turning.

The cutting-force ratio reflects both the friction coefficient on the rake surface and the shear angle in the primary deformation region during the metal-cutting process. According to the cutting-force theoretical model, the friction angle can be obtained from the cutting-force ratio, according to the following formula:

(3) $$\frac{F_{y}}{F_{z}}=\tan\left(\beta -\gamma \right)$$

where *β* represents the friction angle, and *γ* represents the rake angle. Since the rake angle was 0° in this work, the friction coefficient tan *β* is directly equal to the cutting-force ratio. Based on the Merchant metal-cutting principle [26], the shear angle, *φ*, which reflects the chip-deformation degree, can be calculated with the following formula:

(4) $$2\varphi +\beta -\gamma =\frac{\pi }{2}$$

The shear angle and friction angle show the inverse-proportion relationship.

**Figure 9:**
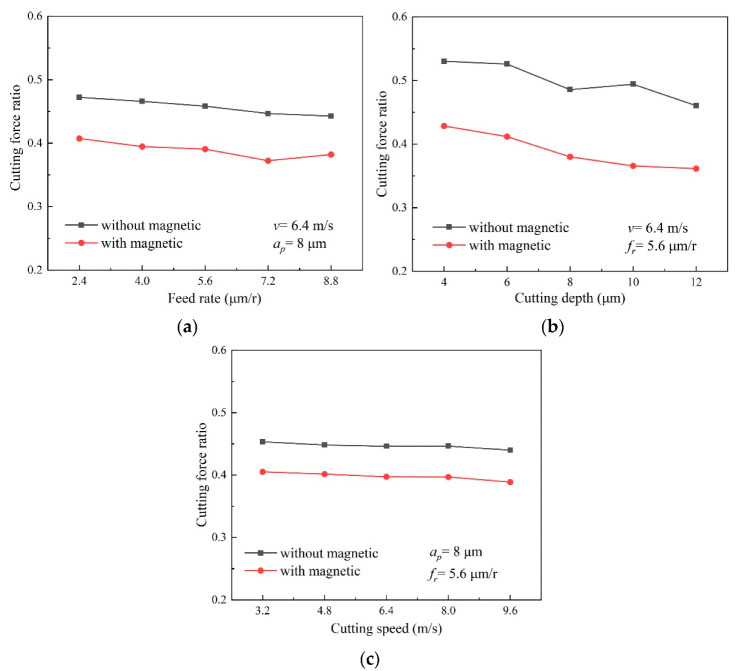
Effect of magnetic-field action on cutting-force ratio, (**a**) cutting-force ratio varying with feed rate, (**b**) cutting-force ratio varying with depth of cut, (**c**) cutting-force ratio varying with cutting speed.

In line with the experiment results, the calculated cutting-force ratio, *Fy/Fz*, under different machining-parameters is depicted in Figure 9. It is revealed that variation trends of the cutting-force ratio both in magnetic-field-assisted turning and the conventional turning, are coincident. The cutting-force ratio slightly decreases along with both the rising depth of the cut and the feed rate, but its overall variation range is very small.

By comparison, the results indicate the cutting-force ratio in magnetic-field-assisted turning is overall much lower than that of traditional turning. Based on the results, the averaged cutting-force ratio becomes approximately 0.39 in magnetic-field-assisted turning, and it is averaged as approximately 0.46 for the conventional turning, without the magnetic field. This indicates that the cutting-force ratio and the friction coefficient reduce by approximately 16% after introducing the magnetic field into the cutting process. Based on the existing research [18,19], the magnetic-field action during the cutting process can improve the tribological properties of the tool/chip contact. This reason is helpful to reduce tool wear, and also explain the reduced cutting-force ratio and friction coefficient in this study. According to the relationship between the friction angle and shear angle from Formula (2), this result also indicates that the shear angle in the cutting process becomes larger, which is helpful for achieving better surface quality.

### 3.3. Chip Morphology

**Figure 10:**
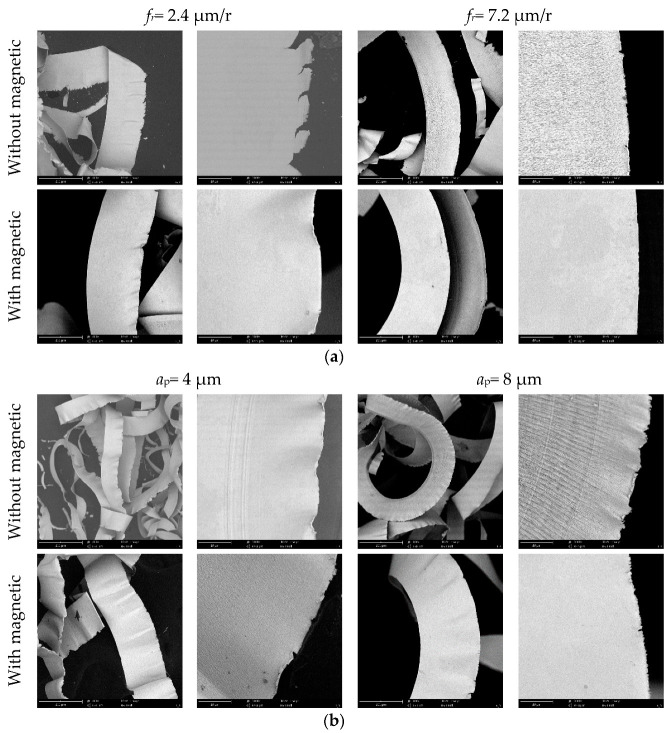

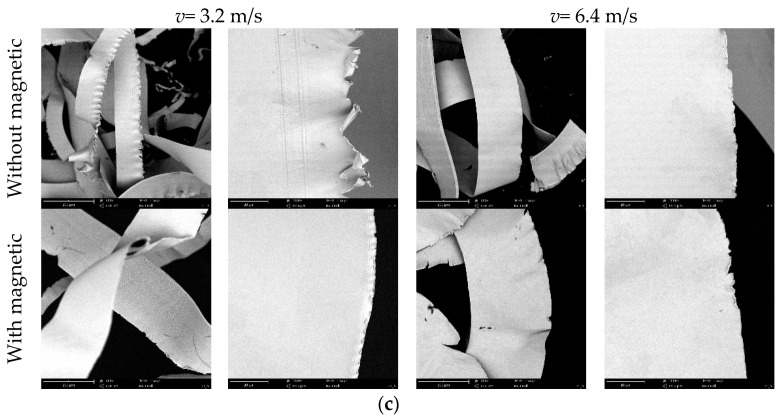
Effect of magnetic-field-assisted turning on chip morphology, (**a**) chip morphology varying with feed rate, (**b**) chip morphology varying with depth of cut, (**c**) chip morphology varying with cutting speed.

The generated chips were collected after the turning experiments. Figure 10 shows the chip morphology comparison from the same magnification view under different machining parameters. Due to the small machining parameters in SPDT, the chip morphology shows thin, flat shape-features. From high magnification images, the side morphology of the chips is curled with features, due to the severe material extrusion and the plastic side-flow in the metal-cutting process [27]. As shown in Figure 10a, with the raising feed rate, the chip width hardly changes. But the curling and tearing features of chip side morphology reduce due to relatively slight chip deformation in metal cutting process. As exhibited in Figure 10b, while the depth of the cut increases, the chip width proportionally increases, due to the larger cutting-width, but the side morphology of these chips shows less change. When the cutting speed gradually rises, the curling and tearing features of the chips’ side morphology greatly decrease, possibly because of the shorter duration time for the workpiece material plastic-deformation in the metal-cutting process, as depicted in Figure 10c.

Through the comparison of chip morphology in magnetic-field-assisted turning and conventional turning, it is found that the chip width becomes larger after introducing the magnetic-field action into the cutting process. Because of the plastic deformation of the formed chips, the chip thickness usually increases and becomes larger than the uncut-chip thickness; however, chip length and width usually decrease and become smaller than the actual cutting distance and cutting width. The chip-compression ratio is calculated by cutting width divided by chip width. From the results, it is revealed that the chip-compression ratio in magnetic-field-assisted turning is smaller than that of the traditional turning. It is known that the friction coefficient on the rake face in magnetic-field-assisted turning is smaller, and this factor helps to obtain the smaller chip-compression ratio. The curling and tearing features of the chip-side morphology obviously reduce in magnetic-field-assisted turning. This results also reveal the lower amount of chip deformation in magnetic-field-assisted turning. According to the literature [28,29], the magnetic-field action can increase the workpiece-material plasticity, based on the magnetoplasticity mechanism, because the magnetic field can promote a dislocation motion in the tensile quality of the material. The better material-plasticity usually leads to the smaller chip-compression ratio in the metal-cutting process, as well. These factors in magnetic-field-assisted turning are helpful for alleviating the severe extrusion and the material plastic side-flow in the metal cutting process, and the trend is to improve metal machinability.

### 3.4. Surface Quality

**Figure 11:**
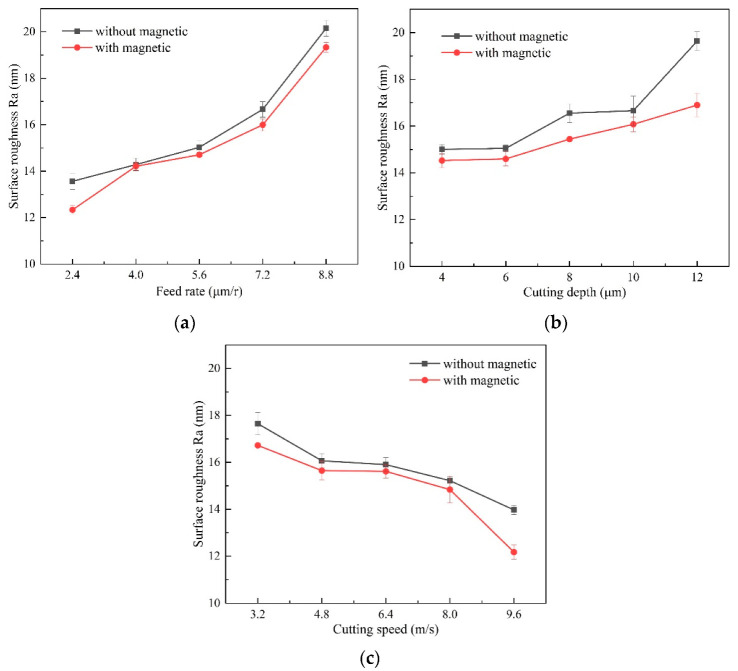
Effect of magnetic-field-assisted turning on surface finish, (**a**) surface roughness varying with feed rate, (**b**) surface roughness varying with depth of cut, (**c**) surface roughness varying with cutting speed.

In the experiments, each surface-roughness result that was measured along the radial direction was repeated five times, in different positions. Figure 11 shows the surface-roughness results under different machining parameters. SPDT can achieve good surface-finish: the obtained surface-roughness in this work varies, in the range of 10–20 nm. With the feed-rate rise, the surface-roughness curve increases slowly at first, and then increases rapidly after the feed rate becomes larger than 5.6 μm/r. Surface roughness gradually rises with the larger depth of cut, and gradually decreases as the cutting speed rises. 

From the surface-roughness comparison in traditional and magnetic-field-assisted turning, it is clear the surface roughness that is achieved in magnetic-field-assisted turning is relative lower than that of the conventional turning because of the smaller friction coefficient and the lower amount of chip deformation during the cutting process. The surface roughness reduces on average from 16.2 nm to 15 nm after introducing the magnetic-field effect into the cutting process. This indicates that magnetic-field-assisted turning is helpful for further enhancing the machined-surface finish. 

**Figure 12:**
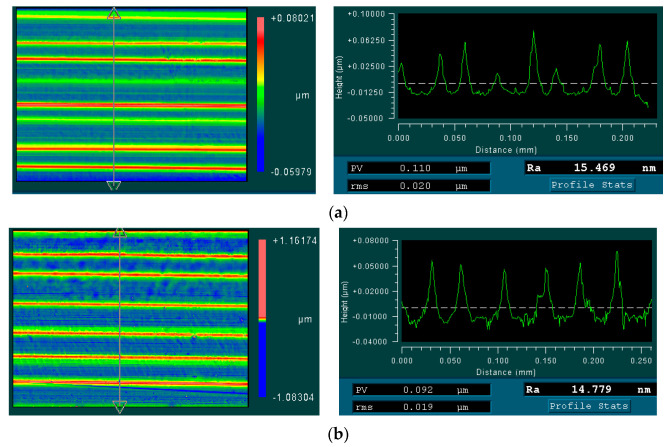
Magnetic-field-assisted turning on surface morphology and profile, (**a**) surface morphology and profile without magnetic, (**b**) surface morphology and profile with magnetic.

Figure 12 shows the typical surface morphology and profile comparison that was obtained in magnetic-field-assisted turning and conventional turning. The periodic cutting marks that were formed by the tool-feed motion are the main feature on the machined surface. In comparison, it is found that the obtained surface-morphology and profile presents the relatively better uniformity of tool marks in magnetic-field-assisted turning compared with that of conventional turning, due to the lower friction-coefficient and less chip deformation in the cutting process. According to the previous research [22], magnetic-field-assisted cutting can reduce the cutting vibration and cutting-force fluctuation during the cutting process; this effect can also improve the uniformity of the cutting marks on the workpiece surface, and achieve a better surface-quality. 

## 4. Conclusions

This work presents an experimental investigation on magnetic-field-assisted turning of single-crystal copper material with a single-point diamond cutting-tool. According to the experiment results, the following conclusions were drawn:

1. Magnetic-field action with magnetic-flux density of 0.38 T is introduced into a single-point diamond-turning system by neodymium iron magnets, to carry out magnetic-field-assisted turning. The cutting force in the magnetic-field-assisted turning increases up to 1.6 times more than the conventional turning because of the additional Lorentz force, which is induced by the equivalent conductor which cuts the magnetic-induction line during the metal-cutting process. Compared with conventional turning, magnetic-field-assisted turning can decrease the cutting-force ratio and friction coefficient on the rake face by approximately 16%, because of the modified tribological property on the tool/chip contact.
2. The chip-compression ratio in magnetic-field-assisted turning is smaller than the conventional turning. Magnetic-field-assisted turning tends to obtain continuous-chip-side morphology with a lower level of chip deformation, due to the magnetoplasticity mechanism. Magnetic-field-assisted turning is helpful for improving metal machinability with friction reduction and the magnetoplasticity effect, the average surface-roughness reduces, from 16.2 nm to 15 nm.

## Figures and Tables

**Table 1:** Single-point diamond-turning parameters.

Parameters	Value
Cutting speed *v* (m/s)	3.2, 4.8, 6.4, 8.0, 9.6
Depth of cut *a_p_* (μm)	4.0, 6.0, 8.0, 10.0, 12.0
Feed rate *f_r_* (μm/r)	2.4, 4.0, 5.6, 7.2, 8.8

## Data Availability

The data that support this study are available within this article.

## References

[1] Zhang S.J., To S., Wang S.J., Zhu Z.W. (2015). A review of surface roughness generation in ultra-precision machining. Int. J. Mach. Tools Manuf..

[2] Shahrokh H., Khaled A.E.H. (2020). Review of non-conventional technologies for assisting ultra-precision single-point diamond turning. Int. J. Adv. Manuf. Technol..

[3] Dai Y., Jiang J., Zhang G., Luo T. (2021). Forced-based tool deviation induced form error identification in single-point diamond turning of optical spherical surfaces. Precis. Eng..

[4] Zhang Z., Yan J., Kuriyagawa T. (2019). Manufacturing technologies toward extreme precision. Int. J. Extreme Manuf..

[5] Liu H.T., Zhu X.F., Sun Y.Z., Xie W.K. (2017). Evolution of stacking fault tetrahedral and work hardening effect in copper single crystals. Appl. Surf. Sci..

[6] Lee W.B., To S., Cheung C. (2000). Effect of crystallographic orientation in diamond turning of copper single crystals. Scr. Mater..

[7] Zhang P., Cao X., Zhang X., Wang Y. (2021). Effects of cutting parameters on the subsurface damage of single crystal copper during nanocutting process. Vacuum.

[8] Yang Z., Zhu L., Zhang G., Ni C., Lin B. (2020). Review of ultrasonic vibration-assisted machining in advanced materials. Int. J. Mach. Tools Manuf..

[9] You K., Yan G., Luo X., Gilchrist M.D., Fang F. (2020). Advances in laser assisted machining of hard and brittle materials. J. Manuf. Process..

[10] Sachin R.P., Phaneendra K.C. (2019). A review of magnetic-assisted machining processes. J. Braz. Soc. Mech. Sci. Eng..

[11] Muju M.K., Ghosh A. (1977). A model of adhesive wear in the presence of a magnetic field. Wear.

[12] Muju M.K., Ghosh A. (1980). Effect of a magnetic field on the diffusive wear of cutting tools. Wear.

[13] Kumagai K., Suzuki K., Kamiya O. (1993). Study on reduction in wear due to magnetization. Wear.

[14] Fei H., Wu H., Yang X., Xiong J., Zhang L., Chen Z., Jiang K., Liu J. (2021). Pulsed magnetic field treatment of cBN tools for improved cutting performances. J. Manuf. Process..

[15] Zhang L., Guo X., Zhang K., Wu Y., Huang Q. (2020). Enhancing cutting performance of uncoated cemented carbide tools by joint-use of magnetic nanofluids and micro-texture under magnetic field. J. Mater. Process. Technol..

[16] El Mansori M., Pierron F., Paulmier D. (2003). Reduction of tool wear in metal cutting using external electromotive sources. Surf. Coatings Technol..

[17] El Mansori M., Iordache V., Seitier P., Paulmier D. (2004). Improving surface wearing of tools by magnetization when cutting dry. Surf. Coatings Technol..

[18] Mkaddem A., Benabou A., El Mansori M., Clénet S. (2013). Analytical modeling to predict the cutting behavior of ferromagnetic steels: A coupled magnetic–mechanical approach. Int. J. Solids Struct..

[19] Alireza D., Sasan K.A., Alireza F.T., Aminollah M. (2017). Effects of magnetic assistance on improving tool wear resistance and cutting mechanisms during steel turning. Wear.

[20] Yip W.S., To S. (2017). Tool life enhancement in dry diamond turning of titanium alloys using an eddy current damping and a magnetic field for sustainable manufacturing. J. Clean. Prod..

[21] Yip W., To S. (2017). Reduction of material swelling and recovery of titanium alloys in diamond cutting by magnetic field assistance. J. Alloy. Compd..

[22] Yip W.S., To S. (2019). Reduction of tool tip vibration in single-point diamond turning using an eddy current damping effect. Int. J. Adv. Manuf. Technol..

[23] Yip W.S., To S. (2019). Control of the ductile and brittle behavior of titanium alloys in diamond cutting by applying a magnetic field. Sci. Rep..

[24] Shaw M.C. (2005). Metal Cutting Principles.

[25] Hatefi S., Abou-El-Hossein K. (2022). Experimental investigation on the effects of magnetic field assistance on the quality of surface finish for sustainable manufacturing of ultra-precision single-point diamond turning of titanium alloys. Front. Mech. Eng..

[26] Hatefi S., Abou-El-Hossein K. (2022). Review of magnetic-assisted single-point diamond turning for ultra-high-precision optical component manufacturing. Int. J. Adv. Manuf. Technol..

[27] Wu X., Du M., Shen J., Jiang J., Li Y., Liu L. (2021). Experimental research on the top burr formation in micro milling. Int. J. Adv. Manuf. Technol..

[28] Ji P.Y., Wang J.A., Ren Z.M., Wang J., Zhang Y.F. (2020). Effect of crystal orientation on magneto-plasticity of mono-crystalline aluminium. Shang Hai Met..

[29] Yip W.S., To S. (2019). Reduction of Minimum Cutting Thickness of Titanium Alloys in Micro Cutting by a Magnetic Field Assistance. IEEE Access.

